# Development and validation of a spectro-temporal processing test for cochlear-implant listeners

**DOI:** 10.1121/1.5079636

**Published:** 2018-11-01

**Authors:** Alan W. Archer-Boyd, Rosy V. Southwell, John M. Deeks, Richard E. Turner, Robert P. Carlyon

**Affiliations:** MRC Cognition & Brain Sciences Unit, University of Cambridge, 15 Chaucer Road, Cambridge CB2 7EF, United Kingdom

## Abstract

Psychophysical tests of spectro-temporal resolution may aid the evaluation of methods for improving hearing by cochlear implant (CI) listeners. Here the STRIPES (Spectro-Temporal Ripple for Investigating Processor EffectivenesS) test is described and validated. Like speech, the test requires both spectral and temporal processing to perform well. Listeners discriminate between complexes of sine sweeps which increase or decrease in frequency; difficulty is controlled by changing the stimulus spectro-temporal density. Care was taken to minimize extraneous cues, forcing listeners to perform the task only on the direction of the sweeps. Vocoder simulations with normal hearing listeners showed that the STRIPES test was sensitive to the number of channels and temporal information fidelity. An evaluation with CI listeners compared a standard processing strategy with one having very wide filters, thereby spectrally blurring the stimulus. Psychometric functions were monotonic for both strategies and five of six participants performed better with the standard strategy. An adaptive procedure revealed significant differences, all in favour of the standard strategy, at the individual listener level for six of eight CI listeners. Subsequent measures validated a faster version of the test, and showed that STRIPES could be performed by recently implanted listeners having no experience of psychophysical testing.

## I Introduction

Although many cochlear implant (CI) listeners understand speech well in quiet, performance varies markedly across listeners and even the most successful struggle in noisy situations. Accordingly there is great interest in developing ways of increasing the number of patients who benefit significantly from a CI, and to improve speech perception in noise; these methods include novel processing strategies (Loizou *et al.*, 2000; Riss *et al.*, 2008; van Hoesel *et al.*, 2008), modes of stimulation (Holden *et al.*, 2002; Donaldson *et al.*, 2005; Litvak *et al.*, 2007a; van den Honert and Kelsall, 2007; Arora *et al.*, 2011; Bierer and Litvak, 2016), and audiological fitting methods (Garadat *et al.*, 2012; Noble *et al.*, 2014). An obstacle to evaluating these new developments is that the most obvious and ecologically valid test, which is to measure their effect on speech perception, suffers from an important confound. Specifically, CI users acclimatise to the way they hear speech in everyday life, and learn the relationship between this pattern of electrical stimulation and individual speech segments (Davis *et al.*, 2005; Davis and Johnsrude, 2007). Accordingly, testing a new development using speech may underestimate or obscure its potential long-term benefits, unless the listener is given extensive take-home experience with the new method. This is not only time consuming, but, if the new method is ultimately not successful, can expose the CI user to weeks or months of degraded speech perception.

The effect of long-term learning and familiarity on speech tests made it desirable to have a non-speech test that was less susceptible to the patient’s experience with their everyday strategy. Although performance on such tests might improve with practice, these effects will be similar for all strategies, including the one used in everyday life, because the stimuli are novel to the listener. Therefore the test will not be biased against a particular (or novel) strategy. An important goal was to develop a test that would be sensitive enough to distinguish between processing strategies that, after several months of implant use, would improve or degrade speech perception.

A number of non-speech tests have been proposed (Supin *et al.*, 1994; Henry and Turner, 2003; Litvak *et al.*, 2007b; Won *et al.*, 2007; Drennan *et al.*, 2008; Saoji *et al.*, 2009; Won *et al.*, 2011; Azadpour and McKay, 2012; Aronoff and Landsberger, 2013). They have, with some notable exceptions, usually been evaluated by correlating performance with speech tests across subjects. This method of evaluation may have some important drawbacks. Clinically, although it is useful to tell a patient which strategy or stimulation method will work best for them, it is less useful to tell them how well they will do compared to other users. Scientifically, it is not clear what value of correlation would correspond to the best evidence in favour of the new method. This is because performance on speech tests would be expected to be influenced by cognitive abilities (Akeroyd, 2008; Mattys *et al.*, 2012; Kaandorp *et al.*, 2017), and by the extent to which the listener has successfully familiarised themselves with their CI (e.g., Fu *et al.*, 2002), but one would hope that a non-speech test would be much less sensitive to these factors. Here, the first steps are taken toward a within-subject evaluation of a new test by evaluating its sensitivity to spectral and temporal degradation both in normal hearing (NH) and CI listeners.

Ideally, a non-speech test would require the listener to perform the types of auditory processing and comparisons that are important for speech perception, without containing recognisable speech segments that might lead to effects of learning or experience. One desirable characteristic is that the listener should have to make both spectral and temporal comparisons of the stimuli. As an extreme example, a speech processing strategy that presented the same stimulation to all electrodes should result in poor performance on both a non-speech and speech test. A processing strategy that smoothed the input with a very long time constant should also result in similar, poor performance in both speech and non-speech tests. A related point is that it should not be possible to perform the task using some local portion of the stimulus, such as the frequency region served by one electrode, one time segment, or one spectro-temporal block (Narne *et al.*, 2016). Rather, the task should require the listener to extract some higher-order feature of the stimuli to be compared; for example, in the test described in this paper, listeners discriminated between sounds that repeatedly sweep either upwards or downwards in frequency. Additional important considerations are that one should be able to titrate task difficulty so as to obtain a threshold, and that there should be some combination of parameters where the task is easily performed by most CI users. Non-speech tests hold another major advantage as they do not need to be translated into different languages, and even the most extensively validated, multilingual speech tests—such as the Oldenburg sentence test—are not available in every language (Kollmeier *et al.*, 2015).

Many of the non-speech tests that have been developed probe either temporal or spectral processing (but not both), with the majority of studies investigating spectral resolution (Supin *et al.*, 1994; Henry and Turner, 2003; Litvak *et al.*, 2007b; Won *et al.*, 2011; Azadpour and McKay, 2012). As noted above, these tests have generally been assessed by correlating performance with measures of speech perception. A number of studies have reported significant correlations for the detection of amplitude modulation (Cazals *et al.*, 1994; Fu, 2002; Won *et al.*, 2011; Gnansia *et al.*, 2014) and for various tests involving spectral ripples (SRs), whereby an otherwise flat frequency spectrum is modulated by a sinusoid so as to contain regular spectral peaks and dips (Supin *et al.*, 1994, 1997; Supin *et al.*, 1999; Litvak *et al.*, 2007b; Saoji *et al.*, 2009; Anderson *et al.*, 2012; Croghan and Smith, 2018). This latter class of test can be subdivided into SR discrimination, where the signals and standard stimuli differ in SR density (in ripples/octave), depth, or phase, and SR detection tasks that measure the minimum ripple depth needed for discrimination between a SR and a noise with a flat frequency spectrum. As a number of authors have pointed out (Azadpour and McKay, 2012; Aronoff and Landsberger, 2013), these tests are susceptible to the use of a number of potentially confounding cues, including shifts in the highest or lowest audible frequency and differences in loudness. These loudness differences can occur either in local spectral regions or across the whole sound when loudness is calculated by a weighted sum of the loudness in different frequency regions. Aronoff and Landsberger (2013) additionally point out that, even where it is shown that task performance is not dominated by these confounds with existing stimulation methods and processing strategies, the use of local cues may re-appear with the new modes of electrical stimulation, for example, focused stimulation methods that one may want to evaluate (Litvak *et al.*, 2007a). It is therefore beneficial to use a test where such cues are unavailable.

Spectro-temporal tests require participants to perform both spectral and temporal comparisons in order to successfully discriminate or detect differences in the presented stimuli. They can also reduce the availability of the local cues found in the spectral- or temporal-only tests.

**Fig. 1:**
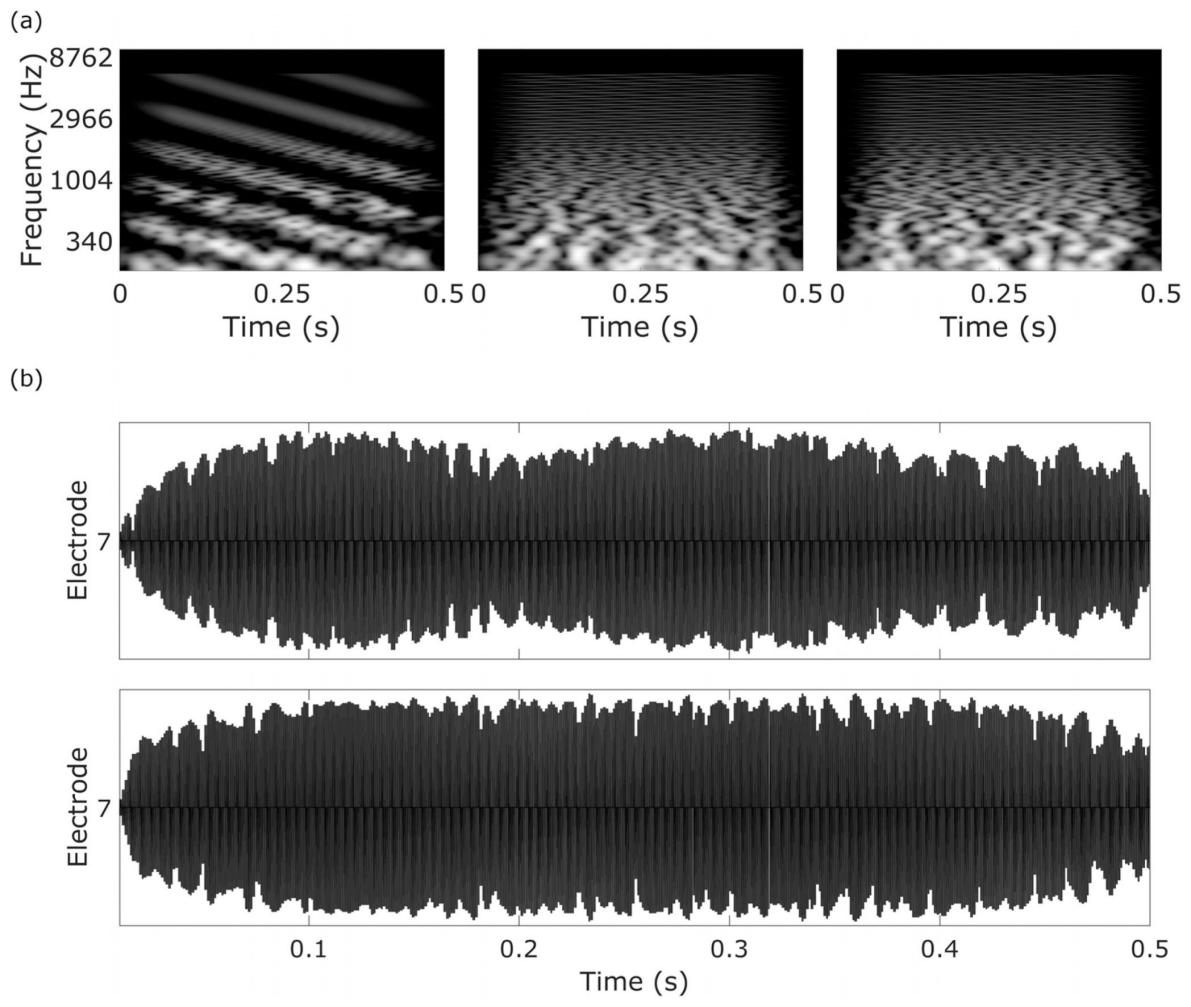
(a) Spectrograms of a SMRT trial showing target (1 RPO, left) and reference (20 RPO, middle, right) stimuli, (b) single-electrode electrodogram showing 1 RPO SMRT target (top) and reference (bottom). A difference in amplitude modulation can clearly be seen between the two stimuli. The electrodogram was generated by the BEPS+ algorithm provided by AB and shows the output of electrode 7 with a HiRes-S map.

A well-known example is the spectro-temporally modulated ripple test (SMRT) developed by Aronoff and Landsberger (2013) and illustrated in Fig. 1(a). The test is a three-interval forced choice task. Two intervals contain a reference stimulus with a very high spectral modulation frequency of 20 ripples (spectral amplitude dips, or troughs) per octave (RPO). These ripples are so close together spectrally as to be undetectable, especially with the greatly reduced spectral resolution of CI listeners when compared to NH listeners. The target stimulus starts at a much lower spectral modulation frequency of 0.5 RPO. These ripples can initially be heard clearly as frequency sweeps in a noisy background. The listener must detect a difference between the references and the target. The SMRT stimulus contains SRs with a modulation phase that shifts over time. This means that all frequency regions receive all levels over the stimulus duration, avoiding the confounding factors of local loudness cues and edge effects. However, some confounds in the SMRT test have been identified, whereby listeners might exploit cues other than those that reflect spectro-temporal processing. One confound is that, at low RPOs (<2), the difference in amplitude modulation on a single channel between the reference and target may be sufficient for the listener to perform the task, reducing the need for the listener to make across-channel spectral comparisons. An example output from one electrode for the SMRT stimulus is shown in Fig. 1(b), illustrating the availability of a local temporal modulation cue at low ripple densities, where the target stimulus produces a more modulated output than the standard stimulus. The potential availability of additional cues is also illustrated by a study by Narne *et al.* (2016). They initially found that NH listeners performed better on a spectrotemporal ripple test similar to SMRT than on a simple spectral test. Analysing the spectro-temporal stimuli, they found an amplitude fluctuation at the outputs of simulated gammatone auditory filters having centre frequencies above 6.4 kHz, which could have provided an additional detection cue for the target stimuli. Removing this confounding fluctuation produced thresholds that were almost identical between the SR and the spectro-temporal ripple tests, suggesting that listeners were using this additional cue.

## II Overview of the STRIPES Test

This section describes a test that is designed to meet the criteria described in Sec. I. Specifically, the test be capable of revealing differences between different processing strategies, rather than between subjects; it should not contain recognisable phonemes, yet require similar auditory processing strategies by the listener to perform the task; it should not be possible to do the test based on one *consistent* spectro-temporal segment or on local cues, instead requiring listeners to compare segments across time and frequency; it should be robust to learning and acclimatisation effects; and the difficulty of the test should be easily adjustable, so as to obtain a measurable threshold, and so as to make it easy enough that most CI listeners are able to do some version of the test. All of the experiments used the same basic method, with generally minor modifications and differences that are described for each individual experiment in Secs. III A, IV B 1, V A, and VI A. Section VII A briefly describes a number of preliminary experiments performed during the development of the final version.

**Fig. 2:**
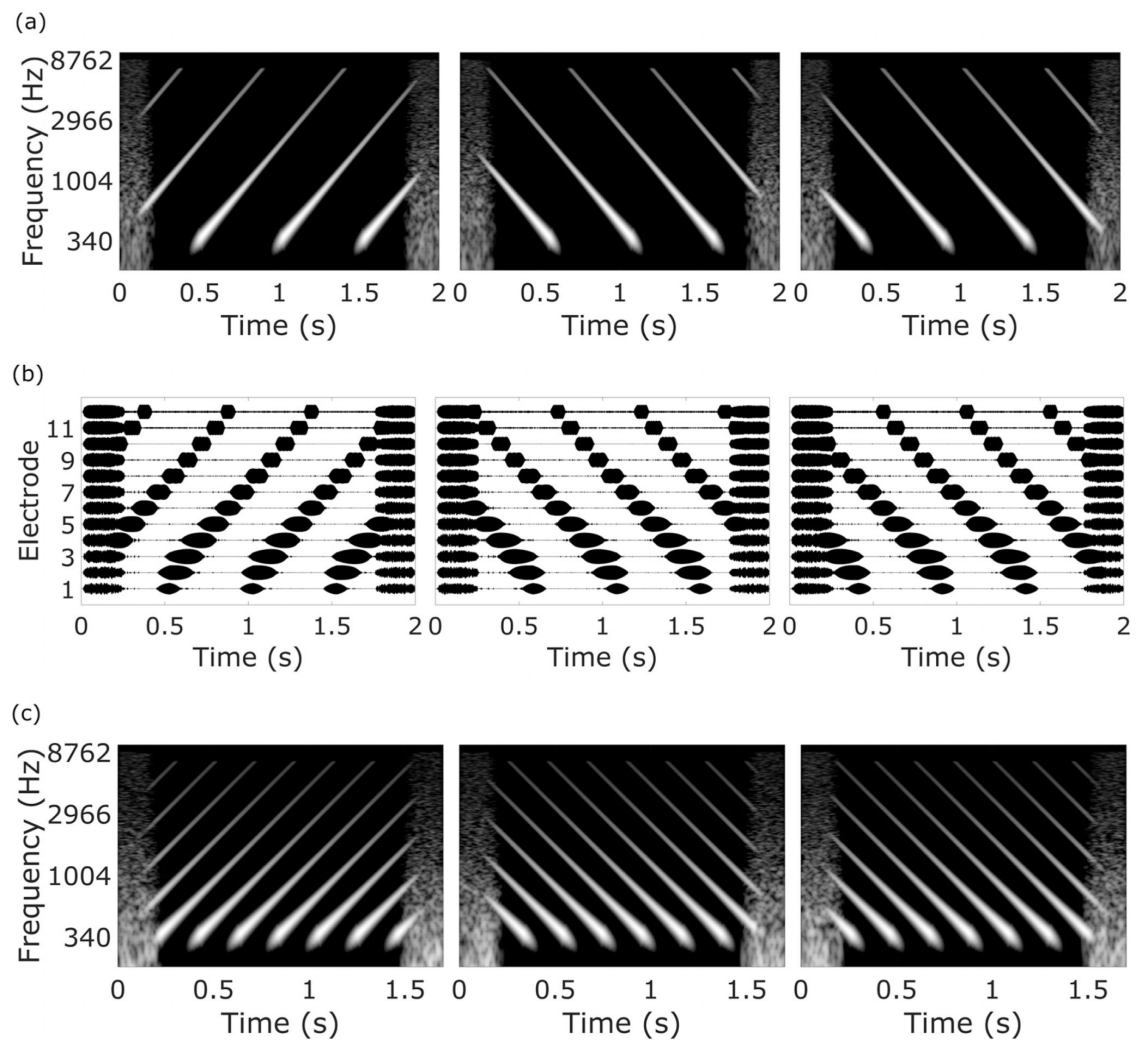
Spectrograms and electrodograms of the STRIPES stimuli. (a) Spectrograms of up (left) and down (middle, right) STRIPES at density = 2, (b) electrodograms of up (left) and down (middle, right) STRIPES at density = 2. (c) Spectrograms of up (left) and down STRIPES (middle, right) at density = 5.

The structure of an example trial of the Spectro-Temporal Ripple for Investigating Processor EffectivenesS (STRIPES) is illustrated in Fig. 2(a). It consists of three consecutive sounds with an inter-stimulus interval of 0.6 s, each of which contains a number of logarithmically spaced sinusoidal frequency sweeps. The listeners are trained to identify and select the interval containing the upward-sweeping sinusoids, which are always the target stimulus. The target is always in either the first or third interval. Pilot studies showed that this version of a three-interval two-alternative forced choice (3I-2AFC) task was time-efficient while producing a good level of performance (see Sec. VII A).

The frequencies of the upward or downward sweeping sinusoids at the start of the stimulus (the starting phase within a cycle of the STRIPES stimuli) are randomised from presentation to presentation. This means that the starting frequencies of the sweeps in each interval are not the same, and so listeners cannot perform the task by comparing the starting frequencies in the three intervals of each trial. Importantly, for any one frequency region, the pattern of amplitude modulation is identical for the upward and downward sweeps. This is shown in the example electrodograms of Fig. 2(b), which correspond to the stimuli of Fig. 2(a). Perceptually, both NH and CI listeners describe the sounds as sweeping up or down in frequency. This higher-order percept allows them to perform the task without having to rely on a local cue. Figure 2(a) also shows the presence of noise bursts at the start and end of each stimulus. The purpose of these “bookends” is to reduce the salience of the onsets and offsets of the stimuli, where, as noted above, the two nonstandard stimuli differ, constituting a misleading local cue. Section VII describes a preliminary experiment that evaluated the effectiveness of the bookends in reducing the use of onset and offset cues.

For the stimuli shown in Figs. 2(a) and 2(b), the density of the frequency glides (Stripes) is equal to 2; at any one instant, two sinusoidal glides are present. The density is also equal to the total number of complete (but not necessarily uninterrupted) glides present during a 1-s interval. To titrate task difficulty the density is varied; an example of a trial with a density of 5 is shown in Fig. 2(c). Increasing density makes the task harder, and either the method of constant stimuli is used to derive a psychometric function relating sensitivity to density, or an adaptive procedure is used that converges on a density where the task can be formed with 70.7% accuracy.

Generation of each STRIPES stimulus involved the summation of a set of 1-s sinusoids, whose frequencies swept exponentially either up or down at the same rate, but with each sweep temporally delayed [Figs. 2(a) and 2(c) show that the sweeps are parallel to one another on a log scale as they increase or decrease in frequency] at a rate of 5 octaves per second. The sine sweeps were produced using code adapted from the Transfer Function Measurement toolbox (Berdahl and Smith, 2008). The temporal spacing between the sinusoids was determined by the desired density. Note that non-integer density values are possible; for example, a density of 2.5 would mean that 50% of the time two swept sinusoids were present simultaneously (overlapped) and that for the other 50% of the time three swept sinusoids overlapped. Each swept sinusoid was turned off when its instantaneous frequency reached 8 kHz (up sweeps) or 0.25 kHz (down sweeps). The duration of the stimulus and of the ramps used to turn the glides on and off differed slightly between experiments and are specified in Secs. III A, IV B 1, V A, and VI A.

The noise bookends at the start and end of each stimulus were constructed in the frequency domain using a method implemented in the “Oscillator and Signal Generator” function (Brimijoin, 2012). The bandwidth of the noise was 100 Hz to 8.7 kHz in order to mask the beginning and end of the STRIPES stimuli in frequency as well as time. The bookends were 250 ms in duration, with raised-cosine onset and offset ramps of 50 and 125 ms, respectively. The end of the noise bookends and the beginning of the STRIPES stimuli were cross-faded together halfway through the ramps.

All stimuli were generated using a sample rate of 48 kHz and presented via a Roland Quadcapture sound card (Roland Corp., Hamamatsu, Shizuoka, Japan) using PsychPortAudio from the Psychophysics Toolbox 3 (Brainard, 1997; Kleiner *et al.*, 2007). Stimulus presentation and response collection were implemented using a custom graphical user interface (GUI) written in MATLAB 2014a (The MathWorks, Inc., 2014).

Sections III, IV, V, and VI each describe one experiment designed to evaluate the STRIPES test. Experiment one provided an initial validation of the STRIPES test using noise-vocoded versions of the stimuli presented to NH listeners. The spectral and temporal sensitivity of the test was measured using an adaptive staircase method. Five conditions were tested: 4-, 8-, 12-, and 16-channel vocoder simulations with a low-pass temporal envelope filter cutoff at 300 Hz, and a 16-channel condition with a low-pass temporal envelope filter cutoff at 3 Hz. Experiment two measured the psychometric functions and adaptive staircase thresholds of CI listeners presented with STRIPES stimuli. The adaptive staircase used the same settings as those used in experiment one. The STRIPES stimuli were altered to reduce the saliency of local, single channel cues. Two 12-channel experimental maps were used to test the spectral sensitivity of the STRIPES test. The standard map used bandwidths similar to those implemented by the clinical software when 12 electrodes are activated, whereas the “blurred” map greatly increased the spectral overlap between each channel. Experiment three reduced the number of step sizes used in the adaptive track from 3 to 2 in order to produce a faster, more clinically useful test that yielded thresholds that were not significantly different from the results of experiment two. The map used was identical to the standard map used in experiment two. Experiment four tested six newly implanted CI listeners with the adaptive staircase methods used in experiments two and three, again using the 12-channel standard map from experiments two and three.

## III Experiment One: Validation with NH Listeners

Experiment one provided an initial validation of the STRIPES test using noise-vocoded versions of the stimuli presented to NH listeners. Many previous studies have tested NH listeners in vocoder simulations so as to simulate the amount of information available to CI listeners (Shannon *et al.*, 1995; Henry and Turner, 2003; Aronoff and Landsberger, 2013). Vocoder simulations were developed as an acoustic method for simulating CI processing strategies from the initial work by Dudley (1939). The hypothesis was that the STRIPES stimuli, when vocoded, should show similar effects to those seen in vocoder speech studies.

Reducing the number of vocoder channels from 16 to 4 was predicted to reduce performance on the STRIPES test in a similar way to the reduction in speech performance (Dudley, 1939; Shannon *et al.*, 1995; Dorman *et al.*, 1997; Fu *et al.*, 1998; Loizou *et al.*, 1999). A large reduction in the cutoff frequency of the low-pass modulation filter used in vocoders was also expected to reduce performance (Shannon *et al.*, 1995, 2001; Xu *et al.*, 2005).

### A Method

Signals were presented via the left earpiece of a set of Sennheiser HD650 headphones (Sennheiser electronic GmbH & Co. KG, Hanover, Germany), and controlled using custom software written in MATLAB. 50 ms raised cosine ramps were used to turn individual glides on and off. The duration of each STRIPES stimulus (excluding bookends) was 1.25 s plus the duration of three cycles at the density used for that stimulus. Note that the duration of one cycle—defined as the delay between successive frequency sweeps—differed as a function of density. For example, with a density of 2, each cycle was 0.5 ms long and the total duration of the stimulus (excluding bookends) was 2.75 s. The root-mean-square (RMS) presentation level was 70 dB sound pressure level. The level was calibrated by presenting a sine-tone complex with tones at octave intervals from 0.25 to 8 kHz and the same RMS as the STRIPES stimuli through calibrated headphones, and measuring the average output level. Participants were seated in a sound-attenuating booth during data collection and made responses via a mouse.

The STRIPES stimuli were noise vocoded to simulate CI listener performance, using custom MATLAB software. The vocoder filters were Greenwood spaced (Greenwood, 1961) and the envelopes were half-wave rectified. The bandwidth of the vocoder was 250 Hz to 8 kHz to match the STRIPES stimuli. Five vocoder conditions were tested: 4, 8, 12, and 16 channels with a 300-Hz envelope filter (to investigate the effect of a reduction in spectral resolution on performance), and 16 channels with a 3 Hz envelope filter (to investigate the effect of a reduction in temporal resolution on performance). The bandpass filters used were third-order Butterworth and the low-pass envelope filter was a second-order Butterworth.

Each trial consisted of a 3I-2AFC (odd-one out) task in which two of the stimuli were down STRIPES and either the first or last stimulus was the signal (up STRIPES). The listener’s task was to select the interval (first or last) containing the up STRIPES stimulus. An inter-stimulus interval of 0.6 s was used. The test used a two-up, one-down (Levitt, 1971) adaptive track, converging on approximately 71% correct. Each run started at a density of 1.1, and the density was increased after every two consecutive correct responses and decreased after every incorrect response. The change from increasing to decreasing density or vice versa was termed a reversal. Each run ended after 12 reversals, and the step size (density change) was 0.5 for the first two reversals, 0.2 for the next four reversals, and 0.1 for the last six reversals. The threshold density was calculated from the average of the last four reversals. Feedback was provided after each response, and a progress bar (based on the number of reversals) was displayed at the bottom of the screen.

Two thresholds per condition were measured for each participant. The presentation order of the conditions was randomised and roughly counterbalanced for each participant by testing each condition in random order for the first threshold measurement, then reversing the order for the second measurement (e.g., conditions 1, 2, 3, 4, 5, 5, 4, 3, 2, 1). Each threshold took 10 to 15 min to measure.

Each adaptive track began with a “pre-test” presentation of 5 trials at the easiest density (1.1). If the listener identified the correct interval four or more times, they proceeded to the adaptive track. Otherwise they returned to the pre-test until they scored four or more out of five trials.

Prior to the start of the experiment, subjects heard ten trials of the adaptive track in each condition with the correct and incorrect answers highlighted during presentation. This allowed listeners to identify the cue they should be listening for during the experiment, and to hear how the task increased in difficulty as density increased.

### B Listeners

Eight NH listeners were recruited. Ethical approval was obtained from the Cambridge Psychology Research Ethics Committee. The average age of the participants was 28 yr (range: 20–40). As the stimuli were presented monaurally to the left ear, audiograms were measured for participants’ left ears only, using a calibrated Madsen Electronics Midimate 602 audiometer. Four-frequency average (0.5, 1, 2, and 4 kHz) thresholds were below 20 dB hearing level (HL) for all listeners (average = 5.5 dB HL, standard deviation = 5.4 dB HL).

### C Results

**Fig. 3:**
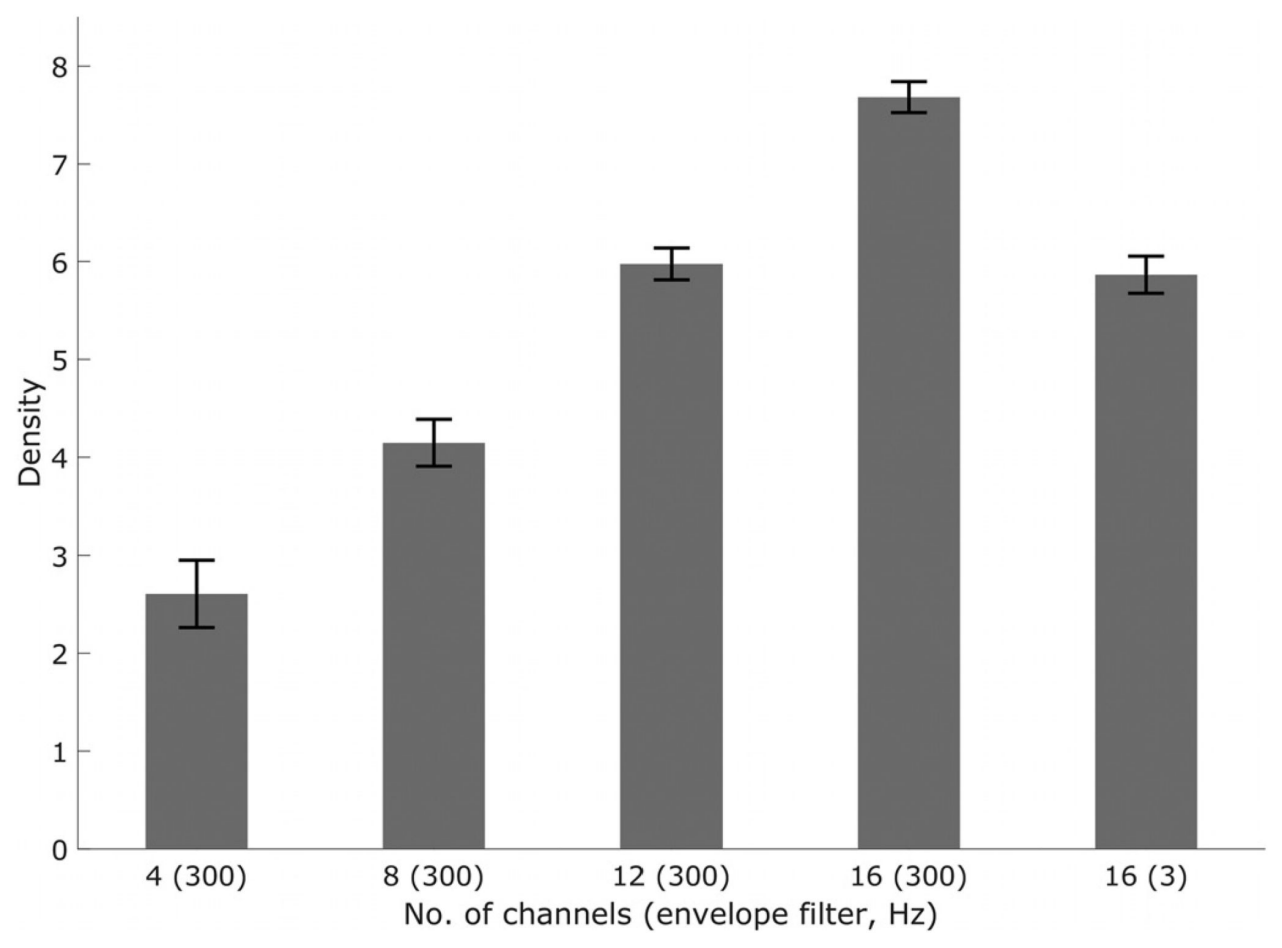
NH vocoder STRIPES mean results from eight listeners. Number of channels (envelope filter) is shown on the abscissa, and STRIPES density (higher equals better performance) is shown on the ordinate. Error bars are the standard error in the mean.

The results of experiment one are shown in Fig. 3. Performance was lowest in the 4-channel vocoder condition, and improved monotonically as the number of channels increased from 4 to 16. Reducing the temporal resolution of the vocoder also impaired performance, and had a similar effect on threshold as reducing the number of channels from 16 to 12. A 1-way repeated-measures analysis of variance (ANOVA) revealed a significant effect of the number of channels for the 4 to 16 channel (300 Hz) results [Greenhouse-Geisser corrected, *F*(1.426) = 217.845, *p* < 0.001]. A two-tailed *t*-test revealed a significant difference between the 16 (300 Hz) and 16 (3 Hz) channel conditions [*t*(14) = 7.32, *p* < 0.001]. The test showed good test-–retest reliability, as all pairs of thresholds measured were different by less than density = 1, with the majority being less than density = 0.5.

The results of experiment one confirm that the STRIPES test is sensitive both to spectral and temporal resolution. Section VII compares these results to the effect of similar manipulations observed in tests of speech perception, and to the results obtained with CI listeners described in Secs. II–IV below.

## IV Experiment Two: Validation of the STRIPES Test using Stimulus Degradation in CI Listeners

**Fig. 4:**
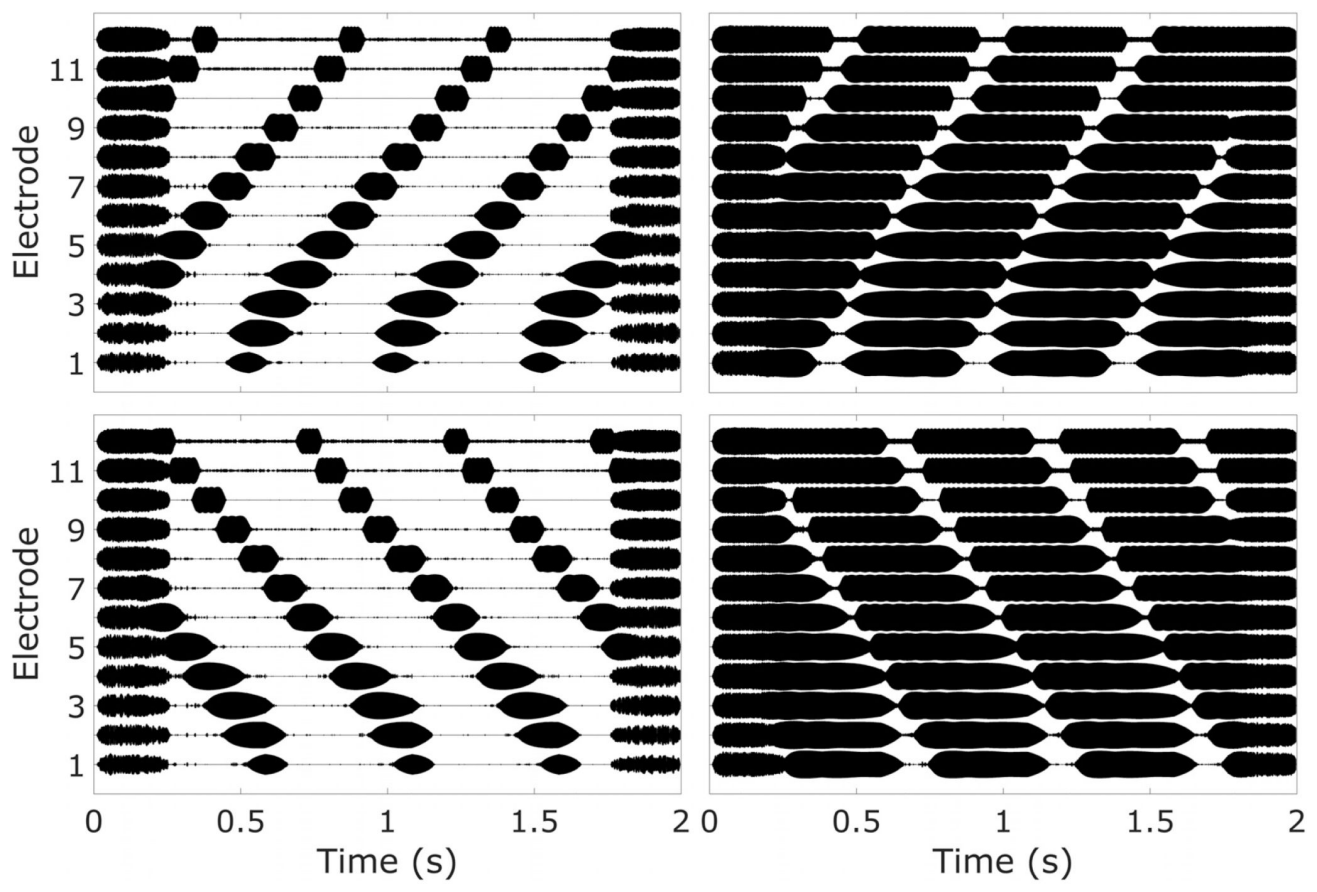
Twelve-channel electrodograms of UP STRIPES (top row) and DOWN STRIPES (bottom row) in no-blur (left column) and blur (right column) conditions at density = 2.

The fact that the STRIPES test is sensitive to spectral and temporal sensitivity in NH listeners with vocoder simulations does not guarantee similar results with CI listeners. Ideally, the STRIPES test would be evaluated using a new processing strategy or method of stimulation that unambiguously improved speech perception. Unfortunately no such method exists yet. Therefore, as an initial test, experiment two evaluated STRIPES by using a manipulation that would be expected to make speech perception substantially worse. Performance on the STRIPES test was measured with a degraded processing strategy and with a “standard” strategy. The degraded strategy roughly simulated the effect of current spread in the cochlea, by increasing the spectral overlap, or “blurring,” between channels. The effect of this on the electrodogram output to the STRIPES stimuli can be seen in Fig. 4 (right) and is discussed in more detail in Sec. IV B. Sensitivity to this degree of spectral blurring would be the minimum expected for a spectrotemporal test to be useful for testing different CI programs.

This type of spectral smearing or blurring would also be expected to reduce speech performance, as shown by the results of several previous NH studies using spectrally smeared 4-channel vocoder simulations (Shannon *et al.*, 1998; Fu and Shannon, 2002; Fu and Nogaki, 2005; Bingabr *et al.*, 2008). These studies generally changed the filter slopes of the frequency channels in order to change the spectral smearing. Because the filter slopes in the Advanced Bionics device were fixed, the present study changed the cutoffs of the filters in order to increase the spectral smearing.

The aims were to test whether the psychometric functions were monotonic for both strategies, and whether the test was sensitive enough to distinguish between strategies on an individual level.

### A Listeners

The demographic information of the CI listeners recruited is given in Table I. All listeners used Advanced Bionics implants. Ethical approval was obtained from the National Research Ethics Committee for the East of England. Before commencing the experiments detailed below, listeners gave their informed consent to participate and were informed that they could withdraw from the study at any point. Subjects were paid for taking part and travel expenses were reimbursed.

### B Method

#### 1 Differences in stimuli compared to NH experiment

The stimuli and method used were similar to those in the NH vocoder study (Sec. III). Here, however, stimuli were presented via the auxiliary (line in) input of a laboratory-owned Research Harmony processor to CI patients. Custom maps were created using the BEPS+ (Bionic Ear Programming System plus) software provided by Advanced Bionics (2014) and downloaded to the processor, which then sent stimulus information and power to the patient’s implant via a standard head coil (Advanced Bionics, Valencia, CA). The CI processor itself added several possible local cues, and it was necessary to use electrodograms to study the output of the processor, and minimize the local cues introduced by various types of processing.

The Advanced Bionics devices contain a de-emphasis filter after the analogue-to-digital converter that is essentially a high-pass filter with a 3 dB cutoff frequency around 1.3 kHz (Boyle *et al.*, 2009). In an extreme case where a CI map used a single-channel, broadband filter, and presented this to one or more electrodes, an increasing (in frequency) exponential sine sweep would produce a different envelope shape to a decreasing sweep. In order to counteract this, an inverse de-emphasis filter was constructed and applied to the STRIPES stimuli (including the noise bookends). The output was inspected using the electrodogram generated by the MATLAB BEPS+ toolbox included with the BEPS+ software. The output from the laboratory owned Research Harmony processor was also passed through a test implant and displayed on a digital storage oscilloscope. The “pre-filtered” stimuli showed a flatter, more symmetric response than the unfiltered stimuli when using a one-channel map.

Inspection of the electrodograms generated by the stimuli of experiment one revealed that, originally, the onset and offset of the amplitude modulations in the lowest and highest frequency channels were asymmetric and the modulations differed in shape. This was caused by the individual sweeps beginning or ending at a cutoff frequency of the filter, and also by the shape of the bandpass filters in the Harmony processor. The lower frequency channels have a shallower frequency roll-off than the higher channels. To correct for this, different-duration ramps were used to turn sweeps on or off when they started or ended at 250 Hz, compared to when they started or ended at 8000 Hz. These ramps were empirically derived by observing the shape of the channel response to a sweep using electrodograms produced using an AB CI simulator included in the MATLAB BEPS+ toolbox. The symmetry of the response was increased by altering the duration and shape of the ramps. The function of the ramp used was: *y* = *m t^b^*, where *m* is a scale factor and *b* = 4 (Scavone, 2004). At 250 Hz the onset (up STRIPES) or offset (down STRIPES) ramp was 100 ms long. At high frequencies, the onset (down STRIPES) or offset (up STRIPES) ramp was 25 ms long. The electrodogram output of a STRIPES stimulus can be seen in Fig. 2(b).

Presentation level was set below the threshold level of the Advanced Bionics automatic gain control (AGC) in order to avoid channel output distortions of the stimuli that could potentially introduce within-channel cues. The maximum level for the soundcard was calibrated using a 1 kHz sine tone with a RMS of 0.1, presented at ±150 mV (peak to peak). An auxiliary cable with built-in attenuation designed for use with the AB ListPlayer speech test presentation software (Advanced Bionics, 2017) was used and the “aux. in” option selected in BEPS+. As an additional check, the output of a clinical Harmony processor was measured in response to STRIPES stimuli at different densities, using the live channel output in the clinical “Soundwave 2.3” software (Advanced Bionics, 2015) combined with a test implant and oscilloscope. Unlike BEPS+ the Soundwave software allows one to turn the AGC on and off, and it was confirmed that this had no effect on the shapes of the output envelopes for levels up to and including the maximum level used in our experiments.

To summarise, the STRIPES test minimised local cues, leading to electrode stimulation patterns that are symmetric for each filter, and where potential loudness cues introduced by the AB pre-emphasis filter were counteracted by passing stimuli through an inverse of that filter. As a result, the only differences between UP and DOWN STRIPES could only be detected via spectro-temporal processing, rather than by the use of spectral or temporal cues alone.

#### 2 Experimental maps

A 12-channel log-linear HiRes-S map (broadly similar to continuous interleaved sampling) was created in BEPS+. The minimum number of active electrodes across listeners was 13. In order to standardize the number of electrodes used across listeners and ensure that all listeners had at least one electrode disabled, 12-channel maps were used. “Fine-structure encoding” was de-selected, as was additional signal processing including “Clear Voice” noise reduction. Pulse width, M, and T levels were identical to those found in each patient’s clinical map. Pulse rate was automatically adjusted in the BEPS+ software to be the same as that set in the clinical Soundwave software. The filter-electrode allocations for this standard “no-blur” map are shown in the left-hand part of Table II.

The effect of current spread in the cochlea was simulated for CI listeners by blurring the output of the channel filters. This was achieved by increasing the width of the analysis filters used from approximately 0.4 octaves used in the logarithmic 12-channel map (“no blur”), to 2 octaves (“blur”), maintaining equal log-linear spacing (e.g., channel 1, 0.25–1 kHz; channel 12, 2–8 kHz). This had the effect of greatly increasing the overlap between channels, artificially broadening the response across the electrode array (Fig. 4, right). The blurring manifested as a longer within-channel output, as each filter was much wider than in the no-blur condition, so each sweep remained in the frequency range of the channel filter for longer. The overlap between filters was also increased, resulting in greater spectral smearing across channels. The filter-electrode allocations for this blur map are shown in the right-hand part of Table II.

#### 3 Procedure

The stimuli were presented at a comfortable level for listeners. This was determined using an 11-point loudness chart provided by AB and routinely used in clinical fitting. Level 6 on this chart was “comfortable,” and our listeners were experienced in using the chart to set levels. The up and down STRIPES stimuli were alternately presented and their level increased on the soundcard initially until the calibrated limit was reached. If a comfortable level was not achieved, the volume control on the Harmony device (which allowed the M level to be varied over a range of ±20%) was used to achieve a comfortable listening level. The comfortable level was bracketed twice by increasing the level until a loudness corresponding to point 7 on the chart (“loud but comfortable”) was reached and then reducing it until level 5 was reached, before finally adjusting it to point 6 on the chart. The standard and blurred maps were loaded into the first and last slots on the research Harmony processor. A copy of the listener’s everyday clinical map was loaded onto the middle program position and used to communicate with the listeners between blocks of trials. Participant AB7 had NH in their non-implanted ear and wore an earplug in that ear during the tests.

Training was provided by presenting the listeners with three repeat trials at five of the densities used during testing (1.1, 2, 3, 4, 5), making 15 presentations in total. The correct answer was highlighted in green and the incorrect answer highlighted in red during the trial. This allowed listeners to become familiar with the stimuli as the task became more difficult. The presentation order of the conditions was counterbalanced across participants.

**Fig. 5:**
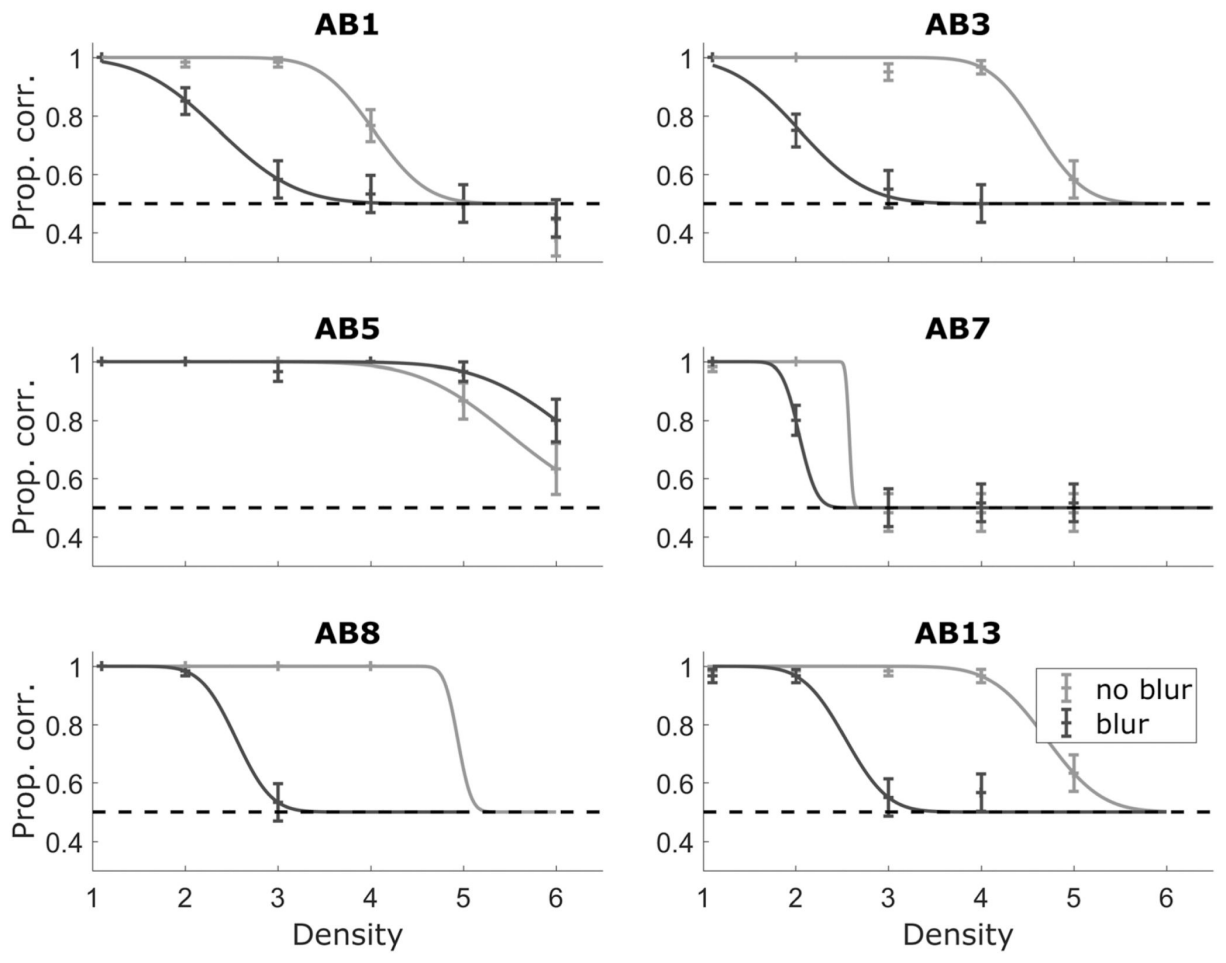
Psychometric functions for a subset (6 of 8) of the CI listeners of experiment two. Error bars show the binomial error. Data points measured using *N* = 30 are: all points for listener AB5, listener AB1 at no-blur and blur density = 1.1, and listener AB8 at no-blur density = 2 (*N* = 30). All other points are *N* = 60.

First, the method of constant stimuli was used to measure psychometric functions. The points used were density = 1.1 (easiest), then increasing integer densities up to a value that depended on listener performance and that ranged between 4 and 6 across listeners. Each data point on the psychometric function was measured with 60 or 30 repeats depending on the time available with the participant and the number of densities measured. The data points measured using 30 repeats are given in the caption of Fig. 5. The psychometric functions were monotonic, justifying the use of an adaptive procedure. An adaptive staircase method identical to that in experiment one was used. All listeners completed three adaptive tracks. The order of the no-blur and blur conditions was randomized across listeners and counterbalanced within listeners.

### C Results

**Fig. 6:**
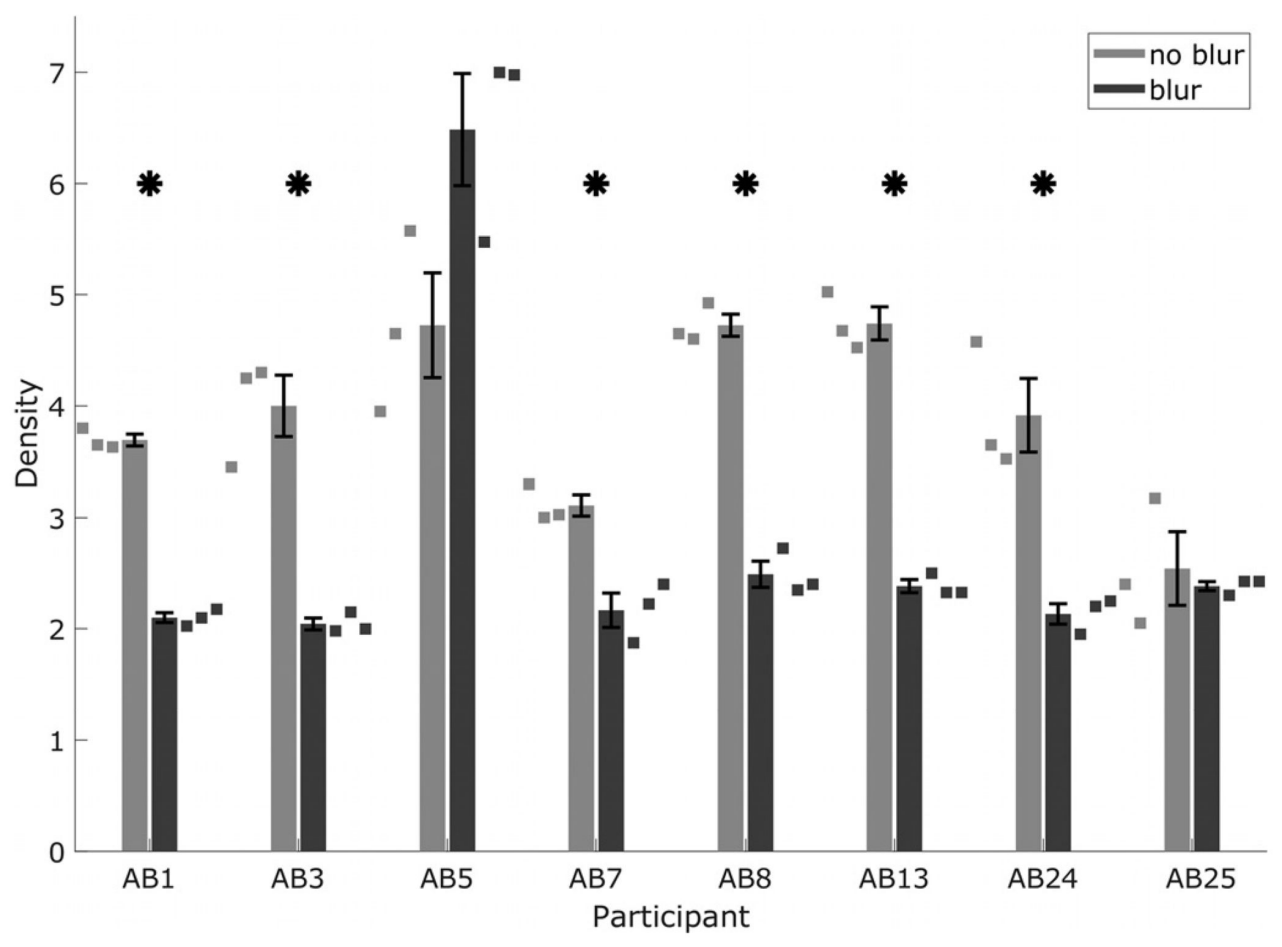
CI STRIPES thresholds for no-blur and blur conditions of experiment two, measured using the original test. Squares show individual runs. “*” denote significant differences between conditions.

Figure 5 shows the individual psychometric functions for a subset of the listeners tested with the adaptive staircase method (Fig. 6). All listeners obtained a score of at least 95% correct at the easiest density in both conditions. All listeners showed monotonic psychometric functions and decreasing performance with increasing density (across the densities measured) in both conditions. Five out of six listeners performed poorly in the blur condition compared to the no-blur condition. Psychometric functions were fitted using a logistic sigmoid and the “fminsearch” function in MATLAB (The MathWorks Inc., 2014). The participant who did not show a difference between the no-blur and blur conditions (AB5) had electrode eight disabled, meaning that their map spanned 13 electrodes, rather than 12. This “break” in stimulation across the array may have provided an additional cue that the listener was able to use. However, further investigations in which subsets of electrodes were disabled indicated that the listener required multiple electrodes spanning a wide range, and not just those near electrode eight, to perform the task. The anomalous results for this listener remain unexplained. However, the poorer performance of most listeners in the blur condition suggested that STRIPES was generally sensitive to large changes in processing strategy.

Figure 6 shows the thresholds obtained using the adaptive staircase method. The thresholds were within 0.5 density of those obtained using the method of constant stimuli for the same listeners in CI experiment one, except for the noblur thresholds of listener AB5 (different by 1 density) and AB3 (different by 0.7 density).

After Bonferroni–Holm correction, two-tailed *t*-tests revealed significant differences between the blur and no-blur conditions for six out of eight listeners, as shown by the asterisks in Fig. 6 and detailed in Table III. All performed better in the standard than in the blurred condition. A oneway repeated-measures ANOVA revealed no significant effect of blurring at the group level. This lack of a grouplevel effect may have been due to the results of listener AB5 who, as in the constant-stimuli procedure, did not show a consistent difference between the blurred and standard map.

The results obtained using the adaptive procedure show that the STRIPES test is sensitive enough to show the effects of large changes in CI processing at the individual level. The individual-run results showed good test–retest reliability, as the difference between the minimum and maximum densities of most listeners’ thresholds was less than 0.5.

## V Experiment Three: A Modified, Faster Version of STRIPES Tested with CI Listeners

Investigation of the adaptive tracks produced during experiment two (Sec. IV) suggested that the density = 0.1 step size was unnecessary for calculation of the threshold, as listeners’ tracks tended to oscillate around the position of the last reversal obtained at density = 0.2. In an attempt to reduce the duration of the test without compromising on the functionality of the adaptive track, a subset of listeners from experiment two were tested on a potentially faster, two stepsize test (modified method) and compared to those collected previously using the previous, three step-size version (original method). The exact duration of a single run of an adaptive staircase procedure can vary due to the nature of the technique.

### A Methods

During experiment two, one participant reported that the noise bookends were louder than the STRIPES stimuli. The RMS value of the noise bookends and STRIPES stimuli were equalized after the inverse de-emphasis filter was applied before presentation through the processor. However, this resulted in a difference in RMS level after the preemphasis filter was applied by the processor. Therefore, for this and subsequent studies, the RMS level of the noise and STRIPES stimuli were set in order to equalize their RMS after the pre-emphasis filter was applied. The input RMS of the STRIPES stimuli remained 0.1. Otherwise the stimuli were the same as those used in the previous study.

The adaptive tracks again used 12 reversals, but this time with two step sizes: 0.5 (4 reversals), and 0.2 (8 reversals). The average of the last four reversals was used to calculate the STRIPES density threshold for one adaptive track.

The stimuli were shortened in order to further reduce the duration of the test. The STRIPES portion of each stimulus was constrained to be more than 1 s long and to contain at least two cycles of the stimulus at a given density. This guaranteed that one uninterrupted sweep was presented regardless of the rove and duration of a cycle. At density = 2, this resulted in the STRIPES portion of the stimulus having a duration of 1.5 s (3 cycles).

Listeners completed at least two runs of the modified test, and training was identical to experiment two (Sec. IV). A subset of the listeners tested in experiment two took part.

### B Results

**Fig. 7:**
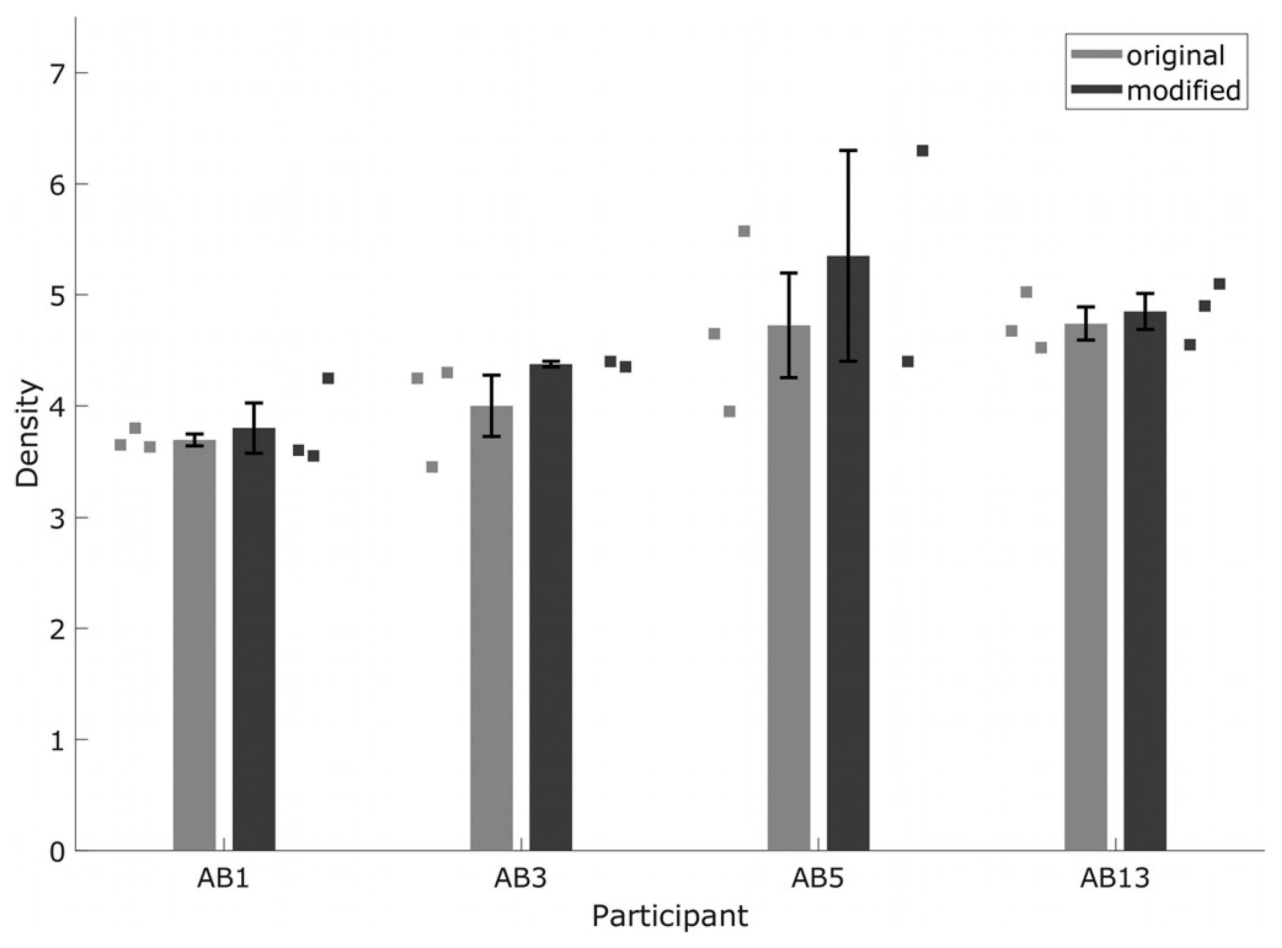
CI STRIPES thresholds for original and modified versions of the test. Squares show individual runs. The original test results are the same as the no-blur condition results shown in Fig. 6. Error bars show standard error of the mean.

The thresholds from the modified method are shown in Fig. 7, which also re-plots thresholds obtained with the original method used in experiment two. Two-tailed *t*-tests revealed no significant differences in performance between the two methods. The results show that a decrease in the number of step sizes and stimulus length did not affect measured thresholds significantly, and reduced the time required to measure one threshold from 15 to 20 min to 5–10 min.

## VI Experiment Four: Testing STRIPES with in Experienced Listeners

An important potential use of the STRIPES test is that it could be applied to newly implanted CI listeners so as to select the best processing strategy or mode of stimulation for each individual user. However, the listeners in experiments two and three were highly experienced with their implants and most had some experience with psychophysical experiments in our laboratory. Therefore six listeners were recruited with less than 1 years’ experience with their implant and no experience with psychophysical experiments. Their thresholds were measured using the standard and modified methods in order to investigate the performance of recently implanted listeners on the STRIPES test. The modified test differed from the original in terms of stimulus length (modified was shorter), number of step sizes (modified used two), and noise bookend level (RMS equalized post preemphasis filter).

### A Methods

The methods for the original and modified tests were the same as in experiments three and four, respectively. As participant AB18 had only 11 active electrodes, the frequency range of channel 11 was extended to cover the same range as channels 11 and 12 in the no-blur map (4520 to 7986 Hz). The listeners were recently implanted (less than 12 months since first activation of their CI). Their demographics are shown in Table IV.

### B Results

**Fig. 8:**
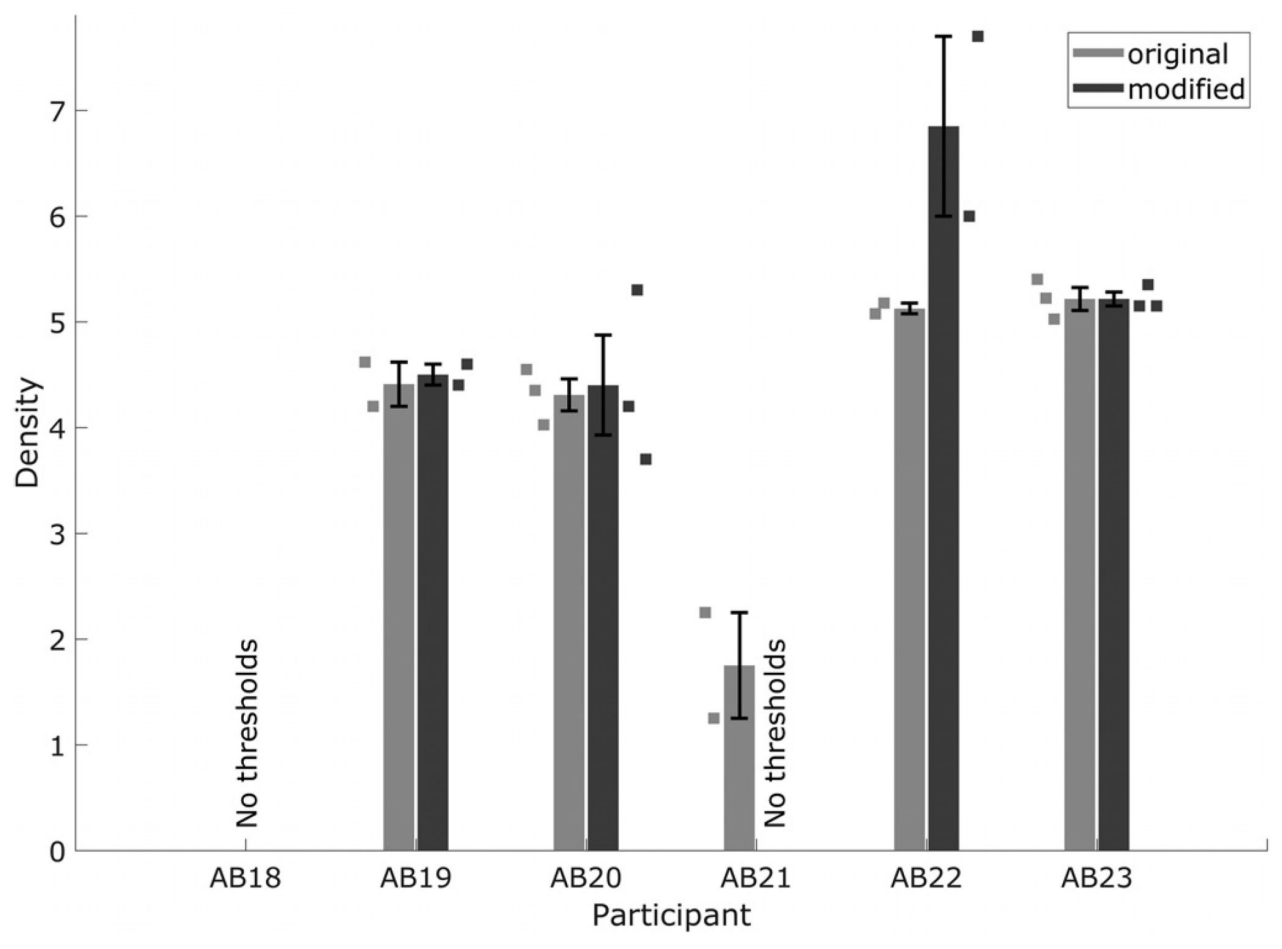
STRIPES thresholds for recently implanted listeners, measured with original and modified tests.

Figure 8 shows that five of the six recently implanted listeners tested were able to complete at least one version of the STRIPES test. The exception was listener AB18, who could not converge on a threshold for either measure. That listener appeared to have very poor speech perception, as communication with the experimenter was possible only via their partner. Listener AB21 was able to converge on a threshold only for the original method. Unfortunately this listener was unable to return for an additional testing session to determine the reasons for this. Thresholds for listeners AB19, AB20, and AB23 were similar for the two methods, and Bonferroni-corrected paired *t*-tests showed no significant differences between the two methods for listeners AB19, AB20, AB22, and AB23. This suggested good test–retest reliability for the STRIPES test. Listener AB22 performed slightly better (higher thresholds) for the modified method, perhaps because the shorter stimuli used in that method reduced the memory load in comparing the three stimuli.

## VII Discussion

### A Development of experimental procedure

The method used for the experiments described here was the result of several preliminary experiments, some of which used a different method of titrating task difficulty. In that method the stripe density was fixed at a value of 2 and task difficulty was manipulated by changing the bandwidth of the Stripes. It was abandoned in favour of the one finally adopted because some CI listeners performed close to chance even with the narrowest Stripes (single sinusoidal glides) at the density of two that was used. Some of the other modifications were based on this earlier design, and this includes the addition of the noise bookends. It was found that when the starting point of the two standard stimuli in each trial was the same, so that participants could use this local cue to identify the target, the bookends decreased performance. Conversely, when these starting frequencies were roved, as in the main experiments described here, the bookends improved performance. It was concluded that starting frequencies provided a local cue; reducing its salience decreased performance when that cue was informative (no rove) and increased performance when it was misleading (rove).

Simulations with NH listeners were also used to compare the 3I-2AFC trial structure employed here with four others: 2I-2AFC (two-interval two-alternative forced choice), 4I-2AFC (four-interval two-alternative forced choice, with two pairs of sounds, in one of which the second member was the target), 3I-3AFC (odd-one-out), and another version of 3I-2AFC in which the target could be the second or third sound (rather than the first or third). Those experiments showed that our eventual 3I-2AFC method yielded the same performance as 4I-2AFC but was faster than it, and that performance was better than with the other methods. This may be because comparisons are easiest when the signal is temporally adjacent to one of the standards. Finally we investigated adaptive procedures (Kesten, 1958; Faes *et al.*, 2007) that, based on simulations and some experimental results (Goldwyn *et al.*, 2010; Barry *et al.*, 2013), have been proposed to be more efficient and/or more resilient to lapses of attention than the standard 2-up-1-down rule proposed by Levitt (1971) and eventually used here. Those simulations showed that, despite its theoretical disadvantages, our 2-up-1-down procedure did not differ from the proposed alternatives either in efficiency or in resilience to lapses of attention, which were simulated by replacing a proportion of the responses with a random choice.

### B Validation using noise vocoder simulations in NH

Experiment one showed that performance on the STRIPES test increased monotonically with increasing numbers of channels and corresponded to densities of 4.0 and 6.0 for 8 and 12 channels, respectively. These thresholds span the range observed for CI listeners with the standard (un-blurred) map in experiment two. Hence, in terms of spectral resolution, the STRIPES test suggests that CI listeners’ performance corresponded to that obtained with 8–12 channels in a NH noise vocoder simulation. This is roughly in line with the conclusions obtained in several studies when comparing NH and CI listeners performing speech tests (e.g., Dorman and Loizou, 1997; Fu *et al.*, 1998; Friesen *et al.*, 2001).

The effects of changing the number of channels is also broadly similar to that observed in speech perception experiments using vocoded tokens, at least for some types of stimulus and presentation methods. As noted above, performance improved monotonically as the number of channels was increased from 4 to 16. A large number of studies have shown that speech perception improves with increases in the number of vocoded channels up to some plateau. The value of the plateau is generally higher for vowels than for consonants, and higher in noise than in quiet. For example, Xu and Zheng (2007) reported a plateau of 12 channels for vowels in quiet and of 16–24 channels for vowels in noise, but a plateau of 12 channels for consonants either in quiet or in noise. It appears from our results that the plateau for STRIPES performance is at or above 16 channels.

Vocoder studies of speech perception have shown that increasing the envelope cutoff frequency improves speech perception up to some value, above which a plateau is observed. The value of that plateau depends on a number of factors, including the number of channels (Shannon *et al.*, 1995; Fu and Shannon, 2000; Souza and Rosen, 2009), the type of speech stimulus, and whether noise or tonal carriers are used (Stone *et al.*, 2008). For example, Xu and Zheng (2007) observed plateaus for the envelope low-pass filter of about 16 and 4 Hz for consonants and vowels, respectively. Experiment one also showed that performance depends on the envelope low-pass filter, but studied only two extreme values of 300 and 3 Hz, with 16 spectral channels. This provided us with a “proof of concept” that STRIPES is sensitive to temporal as well as spectral processing, but does not permit a detailed comparison with data obtained in speech experiments.

### C Limitations of the STRIPES test

As described in Sec. IV, great care was taken to eliminate potential local cues introduced by the Research Harmony processor and/or signal processing strategy used. These changes are likely to be generalizable to changes in stimulation rate and/or pulse shape, and to programming methods that deselect subsets of electrodes, at least when comparing maps with the same number of electrodes deselected. Different modes of stimulation, such as the tripolar and all-polar methods (Litvak *et al.*, 2007a; van den Honert and Kelsall, 2007), may also be investigated without further changes to the stimuli.

Some of the details of the method, such as the deemphasis filter used in experiments two to four, are specific to the Advanced Bionics Harmony processor used and may not generalise to other makes and models. Open source code is available from the authors, and experimenters wishing to use the test with other devices should modify that code accordingly. It is anticipated that, in most cases, the necessary modifications will be minor. As noted in Sec. III, electrodograms that simulated the outputs produced by the processor were examined, and it is recommended that others do likewise both when using STRIPES and other tests. The general point that processors can introduce local and/or spurious cues is not specific to the STRIPES test. For example, O’Brien and Winn (2017) have recently argued that such spurious cues are introduced at high RPOs in the SMRT test, at least for processors manufactured by Cochlear. These cues arise in situations where the spectral density of the stimulus exceeds that of the analysing filters. O’Brien and Winn’s analysis suggested a critical limit of 2.56 RPO on listener thresholds, above which the spectral modulation spectrum obtained from the output of the filterbank did not contain a peak at the RPO used, but did contain other artefacts that could be used to perform the task. Their analysis used 22 filter channels, and our maps used 12. Assuming a linear relationship between channels and performance, our thresholds should not have exceeded approximately 1.4 RPO, which is a STRIPES density of 7. All listeners obtained thresholds well below this limit in the no-blur condition, which was the condition with the most similar filter settings to those used in the O’Brien and Winn study.

Like all spectro-temporal tests developed so far, STRIPES uses a broadband stimulus and so it is not known which frequency region or regions dominate performance. That is, although the test requires spectro-temporal processing over some range of frequency channels, which ranges those have not been manipulated. In NH listeners the concept of the articulation index provides information on which frequency regions are important for speech perception, and if similar information were available for CI listeners then it may be advisable to modify STRIPES so that performance depended more strongly on those regions. Possible methods would be either to test STRIPES multiple times with STRIPES stimuli that begin and end at different frequencies, or to either add noise to or blur the stimulus in some frequency ranges.

Experiment two showed that, for six out of eight listeners, performance dropped significantly when the stimulus was deliberately blurred. However the amount of blurring was substantial and it will be important to know whether the test is sufficiently sensitive to discriminate between more subtle changes. Two preliminary studies from our laboratory produced conflicting results. Carlyon *et al.* (2017) tested subjects who, in a previous study (Bierer *et al.*, 2016), showed performance that differed on some speech tests between standard monopolar maps and two experimental maps that involved more focused stimulation. Neither STRIPES nor SMRT was sufficiently sensitive to reveal an overall difference in performance between the strategies, nor to predict on an individual level which strategy would be best for each listener. However it should be noted that the difference in performance between the strategies was quite small, with a 4% range between the highest and lowest mean scores for 18 participants in a spondee test. Indeed, the variation across maps in the spondee test did not correlate with that in a vowel perception test, so it is perhaps not surprising that STRIPES and SMRT did not correlate with the speech tests. Goehring *et al.* (2018) measured speech performance with patients’ standard clinical maps and in two experimental maps whereby five electrodes were de-activated based on the effects of stimulus polarity on detection thresholds. Subjects differed in which de-activation rule produced the best performance, but STRIPES could, to some extent, predict which map produced the best speech performance in noise for individual subjects. That is, when overall subject differences were removed, there was a significant correlation between STRIPES thresholds and speech-in-noise scores. Predictions based on the results of the SMRT test were not statistically significant.

### D Comparison to other tests

The majority of the thresholds measured across experiments two to four using the standard (un-blurred) map lie in the range of density 3–5, which for a 5-octave stimulus corresponds to 0.6–1.0 RPO. The thresholds measured with SMRT in other labs can be as high as 6 RPO or more (e.g., Aronoff *et al.*, 2016), although recently somewhat lower thresholds have been reported (Goehring *et al.*, 2018). Hence STRIPES typically measures performance for stimuli that are sparser than in the SMRT test. As noted above, dense SRs can interact with CI filter banks and lead to extraneous cues that do not require spectro-temporal processing and the lower spectral densities for STRIPES thresholds make this less likely. In addition, the fact that listeners must identify sweep direction means that any extraneous factors that introduce modulation in one or more channels are less likely to provide a useable cue. These spectral densities also correspond roughly with the range of densities over which performance on static spectral-ripple detection correlates (across subjects) with speech perception (Litvak *et al.*, 2007b).

There are some similarities between the STRIPES test and the Schroeder-phase test developed by Drennan et al. (2008). That test requires listeners to compare complex tones in positive and negative Schroeder phase. The instantaneous frequency of such stimuli are frequency modulated over each period in a saw-tooth pattern, which repeatedly sweeps either up or down for negative and positive Schroeder phases, respectively. Hence, like STRIPES, the Schroederphase test requires listeners to discriminate between repeated frequency sweeps in opposite directions. A substantial difference between the two tests is in the duration of those sweeps, which varies from 20 ms (50 Hz complex) to 2.5 ms (400 Hz complex) in the Schroeder-phase test, compared to 1 s for STRIPES. The rapid channel modulations produced by the sweeps in the Schroeder-phase test may be limited by the same mechanisms that determine the upper limit of temporal pitch in CI listeners (Kong *et al.*, 2009; Kong and Carlyon, 2010). It is also worth noting that no investigation of the effect of the AGC or pre-emphasis filter was reported by Drennan *et al.* (2008). This processing, and/or presentation of the stimuli over a loudspeaker, and detection by a microphone placed above the ear, could have added additional unintended phase distortions to the stimuli. Because Schroeder-phase stimuli have optimally flat envelopes, phase distortions might have resulted in detectable changes in envelope modulation, which could have provided an additional cue.

## VIII Conclusions

The STRIPES test is a spectro-temporal discrimination test designed to investigate CI listeners’ spectro-temporal resolution, while minimizing other cues introduced by the CI processor that could be used to perform the task. The test was validated using vocoder simulations presented to NH listeners using an adaptive staircase method, and was found to be sensitive to changes in spectral (number of vocoder channels) and temporal (envelope filter cutoff) resolution. Monotonic psychometric functions were measured in all six CI listeners tested, and increasing the overlap between analysis filters reduced performance. An adaptive staircase procedure using the same conditions produced similar thresholds. The test was then modified further to reduce the duration of the task and tested on experienced and recently implanted listeners; both groups showed no significant difference in thresholds between the original and modified tests. The test is generalizable to some changes in processing, but, as with all non-speech tests, care should be taken to identify (using electrodograms) and minimize cues that could be introduced to the stimuli due to different forms of CI processing.

## Figures and Tables

**Table I:** Experienced CI listener demographic information for experiments two and three. “Listener” is the unique participant identifier; “Age” is the participant’s age at the time of testing; “Duration of implant use” is the time from the participant’s initial fitting until the date of test; “Onset of hearing loss” identifies the onset and time course of the hearing loss; “Pulse width” is set in the clinical software and used in the research processor; “Deactivated electrodes” are those deactivated in the clinical map.

Listener	Sex	Age	Duration of implant use (years)	Onset of hearing loss	Clinical Processor	Implant type	Pulse width (us)	Deactivated electrodes
AB1	M	71	7	Post-lingual, progressive	Harmony	HR90k/HiFocus 1 J	26.0	16
AB3	M	70	8	Post-lingual, progressive	Na'ida CI Q90	HR 90 k/HiFocus 1 J	19.8	
AB5	M	74	6	Post-lingual, progressive	Harmony	HR90k/HiFocus 1 J	18	8
AB7	F	64	7	Post-lingual	Harmony	HR90k/HiFocus 1 J	21.6	14, 15,16
AB8	F	54	2	Unknown	Na'ida CI Q90	HiFocus MS	35.0	15
AB13	M	84	8	Post-lingual, progressive	Harmony	HR90k/HiFocus 1 J	40.4	
AB24	F	47	1	Post-lingual, sudden	Na'ida CI Q90	HR90k Advantage/HiFocus MS	28.7	
AB25	F	64	1	Peri-lingual, progressive	Na'ida CI Q90	HR90k Advantage/HiFocus MS	18	16

**Table II:** Filter cutoff frequencies for the 12 channel no-blur map and the blur map. The minimum filter cutoff step size in BEPS+ was 68 Hz.

Channel	No-blur map		Blur map	
	Low cutoff (Hz)	High cutoff (Hz)	Low cutoff (Hz)	High cutoff (Hz)
1	238	306	238	986
2	306	442	306	1189
3	442	578	374	1461
4	578	782	442	1733
5	782	1054	510	2141
6	1054	1393	646	2549
7	1393	1869	782	3093
8	1869	2549	918	3772
9	2549	3364	1121	4520
10	3364	4520	1393	5471
11	4520	6015	1665	6627
12	6015	7986	2005	7986

**Table III:** Two-tailed *t*-test results for no-blur and blur conditions in CI experiment two.

Listener	Degrees of freedom	T statistic	*p*
AB1	4	23.11	<0.001
AB3	4	6.97	0.002
AB5	4	−2.55	0.063
AB7	4	5.18	0.007
AB8	4	14.41	<0.001
AB13	4	14.81	<0.001
AB24	4	5.19	0.007
AB25	4	0.47	0.66

**Table IV:** Newly implanted (<1 year) CI listener demographic information for experiment four. Column headings the same as Table I.

Listener	Sex	Age	Duration of implant use (years)	Onset of hearing loss	Clinical Processor	Implant type	Pulse width (us)	Deactivated electrodes
AB18	F	74	<1	Post-lingual, progressive	Naída CI Q90	HR90K Advantage/HiFocus ms	18	12, 13, 14, 15, 16
AB19	M	72	<1	Post-lingual, progressive	Naída CI Q90	HR90k Advantage/HiFocus MS	34.1	
AB20	M	71	<1	Post-lingual, progressive	Naída CI Q90	HR90k Advantage/HiFocus MS	21.6	
AB21	M	57	<1	Peri-lingual	Naída CI Q90	HR90k Advantage/HiFocus MS	40.4	
AB22	F	52	<1	Peri-lingual, progressive	Naída CI Q90	HR90k Advantage/HiFocus MS	36.8	15, 16
AB23	F	57	<1	Peri-lingual	Naída CI Q90	HR90k Advantage/HiFocus MS	24.2	
